# Clinicopathological and Prognostic Role of STAT3/p-STAT3 in Breast Cancer Patients in China: A Meta-Analysis

**DOI:** 10.1038/s41598-019-47556-z

**Published:** 2019-08-02

**Authors:** Yang Li, Yue Wang, Zhixiang Shi, Jinghan Liu, Shuyun Zheng, Jinsong Yang, Yi Liu, Yuhua Yang, Feng Chang, Wenying Yu

**Affiliations:** 1 State Key Laboratory of Natural Medicines, China Pharmaceutical University, Nanjing, 211198 P. R. China; 2 School of International Pharmaceutical Business, China Pharmaceutical University, Nanjing, 211198 P. R. China; 3 School of Life Science and Technology, China Pharmaceutical University, Nanjing, 211198 P. R. China; 4 Nanjing First Hospital, China Pharmaceutical University, Nanjing, 211198 P. R. China; 5 Foreign Languages Department, China Pharmaceutical University, Nanjing, 211198 P. R. China; 6 School of Pharmacy, Guangxi Medical University, Nanning, 530021 P. R. China; 7 The Department of Dermatology, The First People’s Hospital of Changzhou, Changzhou, 213003 P. R. China

**Keywords:** Health care, Oncology

## Abstract

In order to explore the important factors in the diagnosis of breast cancer in China, meta-analysis of previous studies was performed to understand the association between STAT3/p-STAT3 and breast cancer. Information about STAT3/p-STAT3 expression and clinical data about breast cancer in China in particular were gathered from PubMed, Web of Science, CNKI and WanFang databases. RevMan 5.3 and STATA 14.0 were used to analyze the occurrence, development and metastasis of breast cancer for 2818 patients in 18 studies. STAT3/p-STAT3 expression was higher in breast cancer tissue than in normal ones (OR = 7.48, 95% CI = 5.64–9.94), in highly differentiated breast cancer tissue than in lowly differentiated cancer tissues (OR = 2.13, 95% CI = 1.53–2.98), in III/IV stage breast cancer than in I/II stage breast cancer (OR = 3.58, 95% CI = 2.44–5.25), and in tissue with lymphatic metastasis than in normal tissues (OR = 3.72, 95% CI = 2.59–5.35), respectively. Thus, the expression of STAT3/p-STAT3 plays a clinicopathological and prognostic role in the diagnosis and treatment of Chinese breast cancer patients.

## Introduction

Breast cancer remains to be the leading cause of death for women in China. Though the incidence of breast cancer is the highest among all cancers, its early diagnosis is still undesirable^1^. Therefore, it is very necessary to explore the important factors in the diagnosis of breast cancer. Controversial evidence of the relationship between the expression of signal transducer and activator of transcription proteins 3 (STAT3) and breast cancer as a clinicopathologic and prognostic factor in Chinese women has been observed^2^.

STAT3 is a latent cytosolic transcription factor and activates genes in human chromosome 12(q13 to q14–1) by phosphorylation of tyrosine705 in the SH2 domain^3^. Over-activated STAT3 plays an important role in multiple malignant cases, especially in breast cancer^4^. Phosphorylated STAT3 (p-STAT3) dimerizes spontaneously, migrates into cell nucleus and activates the expression of downstream genes to regulate the tumor cell growth, proliferation, differentiation and metastasis^5^. In addition, activated STAT3 was reported to affect the resistance of anti-breast cancer drugs like Paclitaxel^6^ and Adriamycin^7^.

In recent years, due to the complex pathophysiology and various influencing factors, breast cancer, which is usually difficult to be diagnosed and remedied, has become the top lethal cancer for Chinese women^8,9^. A higher STAT3/p-STAT3 expression level was observed in breast cancer tissue than in normal tissues, which aroused our interest to study the relationships between STAT3/p-STAT3 expression level and the occurrence, development and metastasis of breast cancer in Chinese women.

Meta-analysis was used to assess and evaluate the literature reporting the correlations between STAT3/p-STAT3 and breast cancer, with an attempt to decrease the bias of literature and to provide new therapeutic strategy to Chinese women’s breast cancer.

## Materials and Methods

### Search strategy

**Figure 1:**
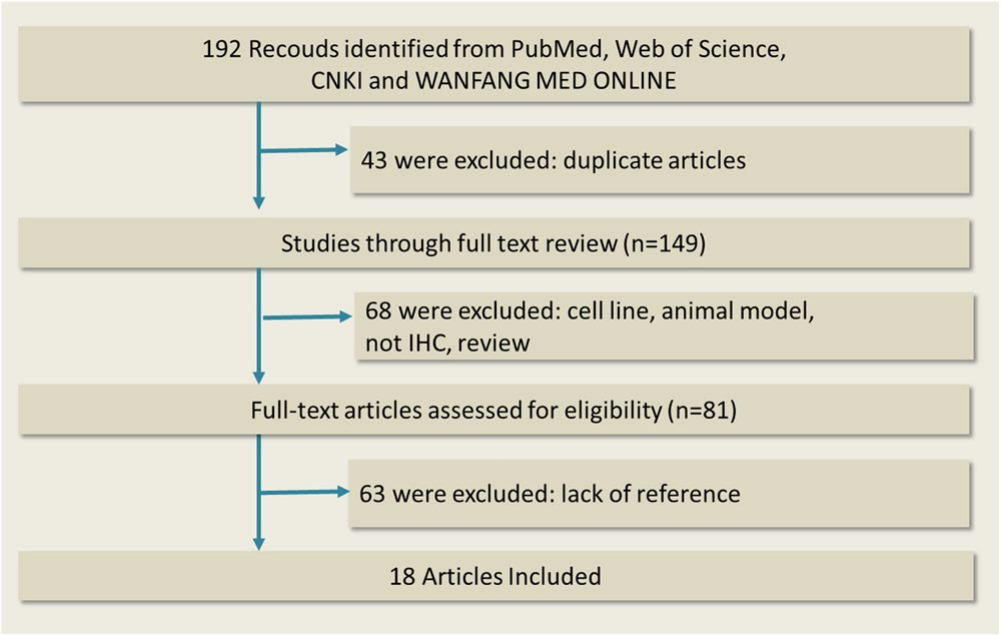
Flow chart of literature search.

PubMed, Web of Science, CNKI and WanFang were used to search for papers concerned, which were published before March 2019. Terms in the searching strategy like “breast cancer”, “breast tumor”, “STAT3 “, “Signal transducer and activator of transcription 3”, “p-STAT3” and “phospho-STAT3” were used. The flow chart of the literature search is shown in Fig. 1 as follows.

### Inclusion criteria

(1) Studies are from journal articles; (2) Normal breast tissue or breast benign hyperplasia as a control group was provided; (3) Immunohistochemistry (IHC) must be used in the studies to detect the expression level of STAT3 and p-STAT3 in the breast carcinoma and non-carcinomatous tissue; (4) The research materials in the studies should be from hospitals.

### Exclusion criteria

(1) Literatures published as letters, reviews or meeting reports; (2) Articles without a contrast with non-carcinomatous tissue; (3) The research materials collected from breast carcinoma cell lines or animal tumor model; (4) Incomplete data.

### Screen and Excerpt

All studies were brought into this research by two researchers independently according to the exclusion and inclusion criteria, with the full text being acquired with the extracted data inside. After all the data were crosschecked, divergence would be discussed and the third researcher would give some references. The extracted data included characteristic data, the focused type, numbers of treatment and control groups and the expression level of STAT3 or p-STAT3 in treatment and control groups, age groups, tissue types, TNM stages, tumor sizes and the states of lymphatic metastasis.

### Quality assessment

Quality assessment was performed by two researchers separately, with differences being resolved through discussion. We referred to the Cochrane evaluation clauses: (1) Random sequence generation; (2) Allocation concealment; (3) Blind method; (4) Incomplete outcome data; (5) Non-selective ending report; (6) Without other bias source. Each coincident item gives one point. Studies with scores ≥3 were assigned as high-quality studies.

### Statistical analysis

Extracted data were used to analyze the correlation between the expression of STAT3 or p-STAT3 and the focused type, the numbers of treatment and control groups, the expression level of STAT3 or p-STAT3 in treatment and control groups, age groups, tissue types, TNM stages, tumor sizes and the states of lymphatic metastasis. All data in literatures were combined to obtain a value of OR (Odds Ratio) and 95% CI (Confidential Interval). For the results of *χ*^2^ test, when P < 0.1 and I² > 50%, supra presence included significant heterogeneity, using the random effects model by choosing the model option in the Review Manager 5.3 software when generating the forest plots. Then, regression was analyzed by drawing funnel plot and Egger’s test. The analysis was performed using STATA statistical software version 14.0.

## Results

### Essential characteristics and quality evaluation

According to the inclusion and exclusion criteria, 2818 tissue samples of 18 research studies were chosen and analyzed. Among them, 1672 cases (1188 breast cancer tissues and 484 normal tissues) of 9 studies focused on expression level of p-STAT3 while 1445 cases (870 breast cancer tissues and 575 normal tissues) of 12 studies focused on expression level of STAT3, as shown in Table 1. Yang Z and Ying MZ’s studies contained both STAT3 and p-STAT3.

**Table 1:** Essential characteristics and quality evaluation in the research.

No.	First author	Year	Type of study	Control group	Experiment group	Score	PA
1	Chen TT^35^	2012	Case control test	36	160	4	Santa Cruz, USA
2	Fang M^36^	2014	Case control test	48	33	4	ab15523, Abcam, UK
3	Guo W^37^	2009	Case control test	30	76	3	MXB, PRC
4	Li SJ^38^	2011	Case control test	25	42	4	Abcam, UK
5	Ma JW^39^	2012	Case control test	40	84	4	ZSGB, PRC
6	Qi FJ^40^	2010	Case control test	30	80	3	MXB, PRC
7	Zhang N^41^	2016	Case control test	24	355	3	NR
8	Wang J^42^	2015	Case control test	245	379	4	Abcam, USA
9	Zhou T^43^	2013	Case control test	15	93	3	sc-99086, CA
10	Xu S^44^	2016	Case control test	160	80	4	Santa Cruz, USA
11	Yang J^45^	2012	Case control test	26	126	4	BA0621, BOSTER, PRC
12	Yang Z^46^	2011	Case control test	10	36	4	MXB, PRC
13	Ying MZ^47^	2007	Case control test	41	71	4	Upstate, USA
14	Yue XC^48^	2009	Case control test	25	51	5	ZSGB, PRC
15	Zhang W^49^	2008	Case control test	12	45	4	CST, USA
16	Wang QT^50^	2017	Case control test	73	57	4	BS1141R, CHN
17	Tan QF^51^	2017	Case control test	41	19	5	Santa Cruz, USA
18	Chen TT^52^	2016	Case control test	50	100	4	Santa Cruz, USA

PA, primary antibody used for IHC; The score is based on cochrane risk of bias tool: 1: adequate sequence generation; 2: allocation concealment; 3: blinding; 4: incomplete outcome data address; 5: free of selective reporting; 6: free of other source of bias.

Results from the 18 research studies: 13 studies reported the correlation of expression level and breast cancer occurrence (6 on p-STAT3 and 7 on STAT3, with Yang Z and Ying MZ’s studies containing both STAT3 and p-STAT3); 13 studies reported the correlation of STAT3 expression level and histological differentiation (5 on p-STAT3 and 8 on STAT3, with Ying MZ’s study containing both STAT3 and p-STAT3); 15 studies reported the correlation of expression level and breast cancer TNM stages (8 on p-STAT3 and 7 on STAT3, with Yang Z and Ying MZ’s studies containing both STAT3 and p-STAT3); 11 studies reported the correlation of expression level and breast cancer lymphatic metastasis (7 on p-STAT3 and 4 on STAT3). The results are shown in Table 2.

**Table 2:** Characteristics of included studies in the research.

No.	First author	Year	Type	Age (+/−)			Lymph node metastasis (+/−)		Histological grades (+/−)			TNM phases (+/−)				Tumor sizes (cm) (+/−)	
							Positive	Negative	1	2	3	1	2	3	4	≤2:	>2:
1	Chen TT	2012	p-STAT3	≤35:13/3	35–55:62/30	>55:36/16	NR	NR	8/5	69/33	34/11	47/39		64/10		2/11	109/38
2	Fang M	2014	STAT3	≤50:18/0	>50:14/1	NR	NR	NR	9/0	12/1	11/0	8/0	14/1	4/0	4/0	NR	NR
3	Guo W	2009	STAT3	≤50:26/9	>50:24/17	NR	32/10	18/16	23/19		27/7	30/22		20/4		17/10	33/16
4	Li SJ	2011	STAT3	≤50:20/17	>50:22/8	NR	32/11	10/14	NR	NR	NR	5/7	18/14	19/4	NR	17/7	25/18
5	Ma JW	2012	p-STAT3	NR	NR	NR	32/14	20/18	23/22		30/9	32/23		22/6		NR	NR
6	Qi FJ	2010	STAT3	NR	NR	NR	34/10	18/18	23/20		29/8	31/23		21/5		NR	NR
7	Zhang N	2016	p-STAT3	NR	NR	NR	159/8	196/16	NR	NR	NR	292/20		63/4		109/113	246/266
8	Wang J	2015	p-STAT3	NR	NR	NR	159/8	196/16	NR	NR	NR	292/20		63/4		109/4	246/20
9	Zhou T	2013	STAT3	≤50:43/4	50:50/11	NR	64/4	21/11	21/3	42/6	30/6	NR	NR	NR	NR	NR	NR
10	Xu S	2016	STAT3	NR	NR	NR	21/12	31/16	3/1	31/11	18/16	NR	NR	NR	NR	NR	NR
11	Yang J	2012	STAT3	<50:50/12	≥50:48/16	NR	76/8	28/14	10/2	58/14	36/6	NR	NR	NR	NR	NR	NR
12	Yang Z	2011	STAT3	≤50:10/7	>50:11/8	NR	12/3	9/12	NR	NR	NR	11/13		10/2		6/7	15/8
			p-STAT3	≤50:9/8	>50:11/8	NR	13/2	7/14	NR	NR	NR	6		11/1		8/5	12/11
13	Ying MZ	2007	STAT3	≤40:11/5	41–60:37/8	>60:8/2	31/9	25/6	5/5	22/6	29/4	6/2	22/12	24/1	4/0	NR	NR
			p-STAT3	≤40:13/3	41–60:28/17	>60:8/2	34/6	15/16	7/3	14/14	28/5	4/4	20/14	23/2	2/2	NR	NR
14	Yue XC	2009	STAT3	<35:7/1	35–50:26/3	>50:11/3	28/1	16/6	10/3	24/2	10/2	7/1	31/5	6/1	NR	6/4	32/1
15	Zhang W	2008	p-STAT3	<50:7/4	≥50:20/14	NR	14/4	13/14	NR	NR	NR	NR	NR	NR	NR	1/3	26/15
16	Wang QT	2017	STAT3	<60	≥60	NR	30/27	8/65	11/8	43/53	3/12	2/9	23/55	32/9	NR		
17	Tan QF	2017	p-STAT3	NR	NR	NR	NR	NR	23/20		15/2	22/21		16/1		15/10	27/8
18	Chen TT	2016	STAT3	≤35:11/4	≤55:58/29	>55:31/17	76/24	22/28	7/5	61/34	32/11	43/36		57/14		2/10	98/40

NR: No report; p-STAT3: phosphorylated STAT3; TNM: tumour node metastases; + :STAT3/p-STAT3 positive; -:STAT3/p-STAT3 negative.

### The results of STAT3 expression and analysis

#### Correlation between STAT3/p-STAT3 expression level and breast cancer occurrence

**Figure 2:**
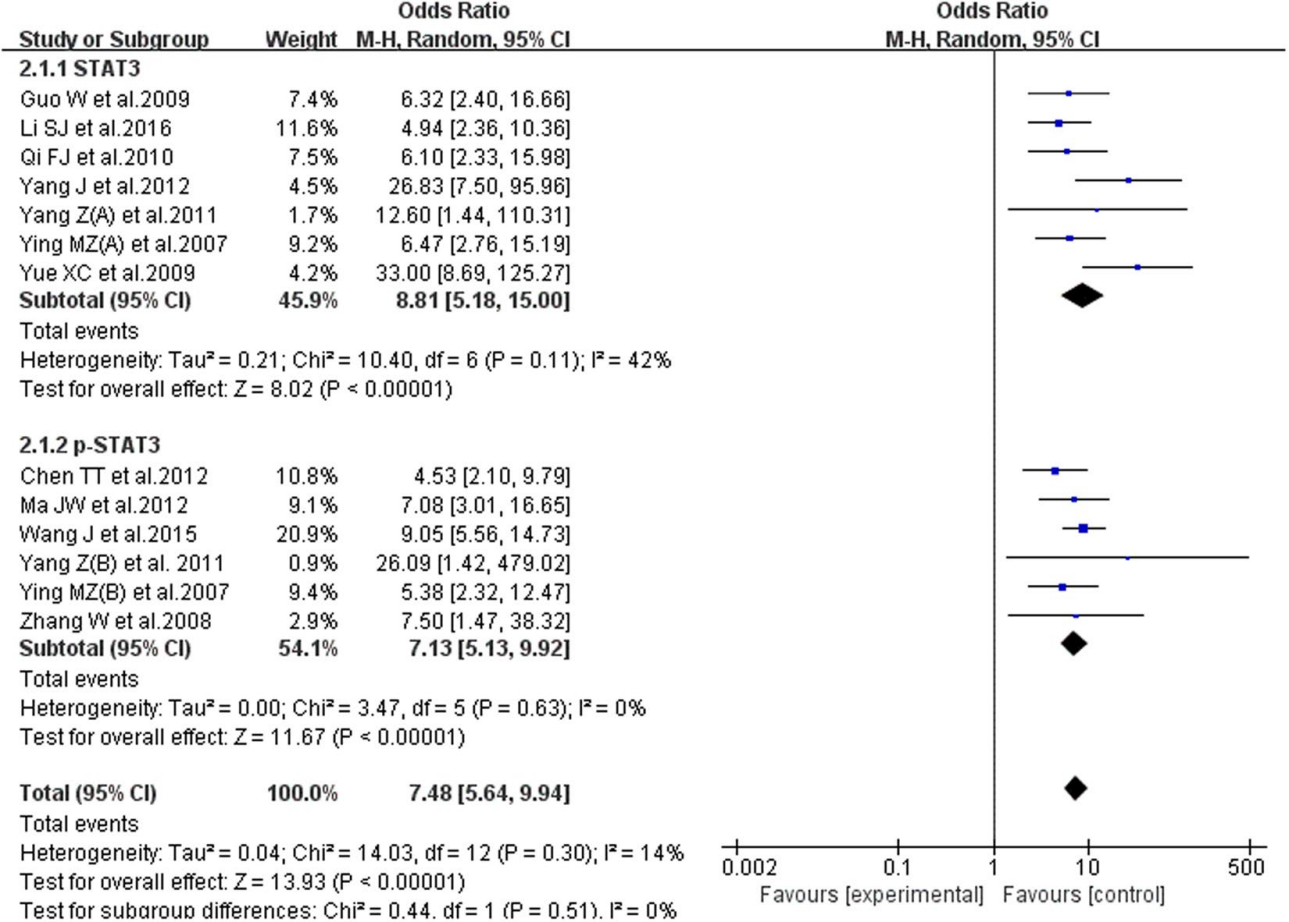
Forest plot of correlation between STAT3/p-STAT3 expression level and breast cancer occurrence. Random-effects OR = 7.48, 95% CI = 5.64–9.94, P = 0.30, I² = 14%.

13 studies reported the correlation between STAT3 expression level and breast cancer occurrence, 6 on p-STAT3 and 7 on STAT3, 1362 cases for breast cancer tissues and 773 for normal tissues. No substantial heterogeneity existed with each group (P = 0.30, I^2^ = 14%), and we performed the meta-analysis using the random-effects model. STAT3/p-STAT3 expression level in breast cancer tissues was higher than that in normal ones (OR = 7.48, 95% CI = 5.64–9.94). In the subgroup analysis, we achieved a consistent result (STAT3: OR = 8.81, 95% CI = 5.18–15.00; p-STAT3: OR = 7.13, 95% CI = 5.13–9.92). The results demonstrated that the STAT3/p-STAT3 expression level in breast cancer tissue was higher than that in normal ones (Fig. 2).

#### The correlation of STAT3/p-STAT3 expression level and histological differentiation

**Figure 3:**
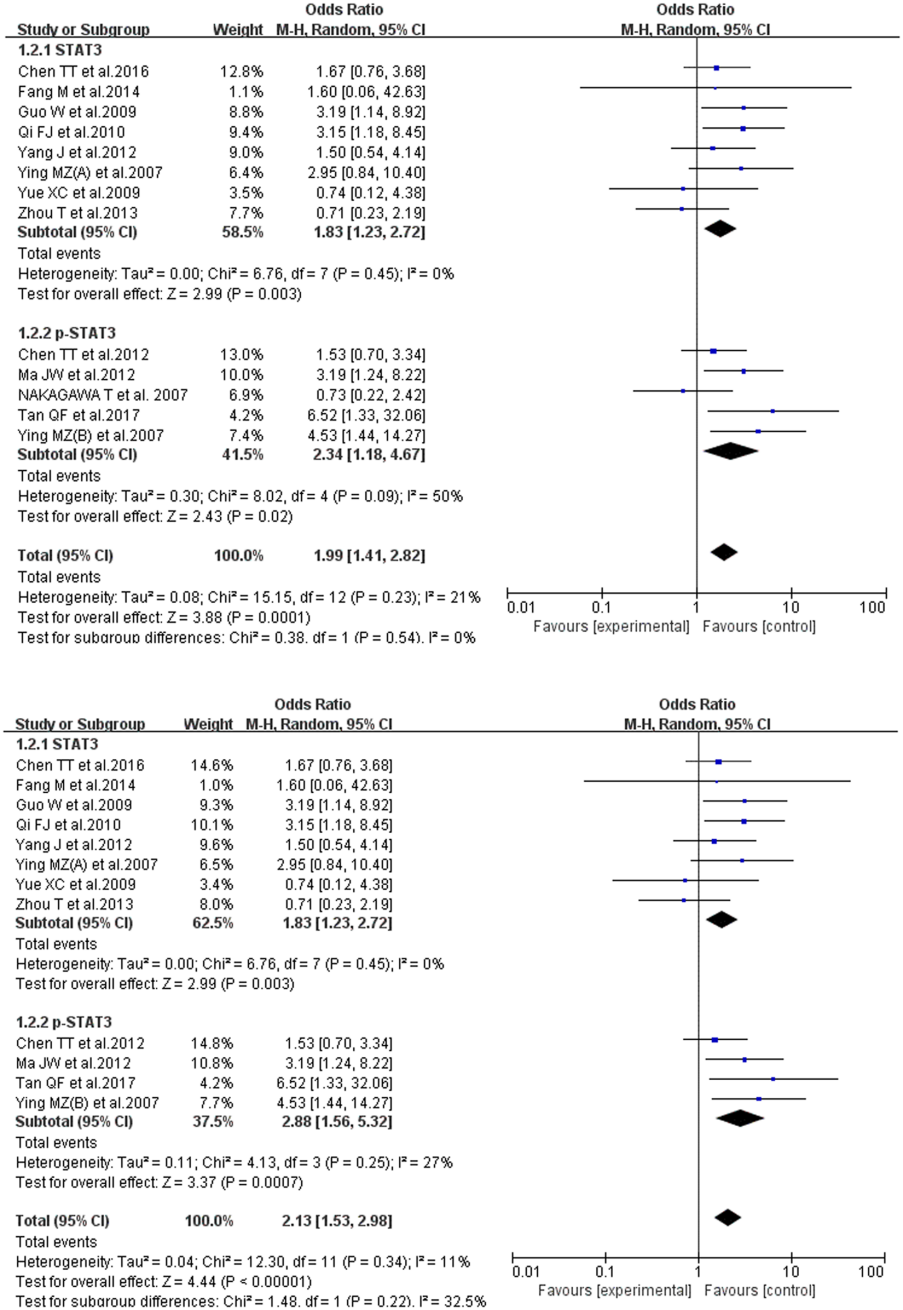
Forest plot of Correlation between STAT3/p-STAT3 expression level and histological differentiation. Random-effects OR = 2.13, 95% CI = 1.53–2.98, P = 0.25, I² = 27%.

13 studies reported the correlation of STAT3/p-STAT3 expression level and histological differentiation, with data inside being used to analyze the difference between low-differentiated and high-differentiated cases. In the 13 reports, 395 cases were high-differentiated and 750 were low-differentiated. There was no substantial heterogeneity in each group (P = 0.25, I^2^ = 27%), and the random-effects model was chosen. The result showed that the STAT3/p-STAT3 expression level in high-differentiated cases was higher than that in low-differentiated cases (OR = 2.13, 95% CI = 1.53–2.98). In the subgroup analysis, we achieved a consistent result (STAT3: OR = 1.83, 95% CI = 1.23–2.72; p-STAT3: OR = 2.34, 95% CI = 1.18–4.67). The analytical results were stable, as shown in Fig. 3.

#### The correlation of STAT3/p-STAT3 expression level and breast cancer TNM stages

**Figure 4:**
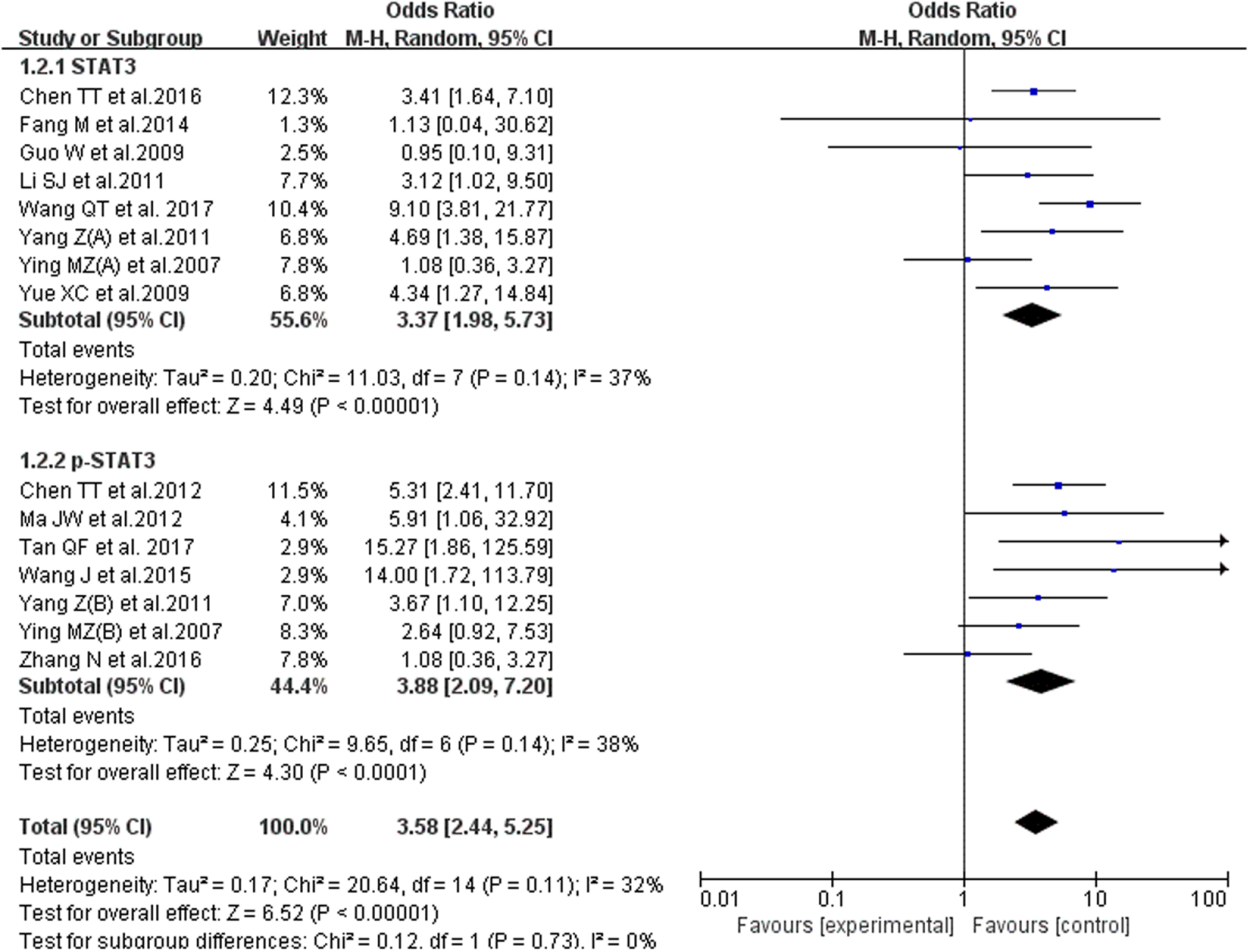
Forest plot of Correlation between STAT3/p-STAT3 expression level and breast cancer TNM stages. Random-effects OR = 3.58, 95% CI = 2.44–5.25, P = 0.11, I² = 32%.

15 studies reported the correlation of STAT3/p-STAT3 expression level and breast cancer TNM stages. We regarded stages I and II as early stage (involving 1271 cases), III and IV as late stage (involving 509 cases). We used the random-effects model and there was no evident heterogeneity inside (P = 0.11, I^2^ = 32%). The results showed that the STAT3/p-STAT3 expression level in breast cancer of the late stage was much higher than the early stage (OR = 3.58, 95% CI = 2.44–5.25). In the subgroup analysis, we achieved a consistent result (STAT3: OR = 3.37, 95% CI = 1.98–5.73; p-STAT3: OR = 3.88, 95% CI = 2.44–5.25), as shown in Fig. 4.

#### The correlation of STAT3/p-STAT3 expression level and breast cancer lymphatic metastasis

**Figure 5:**
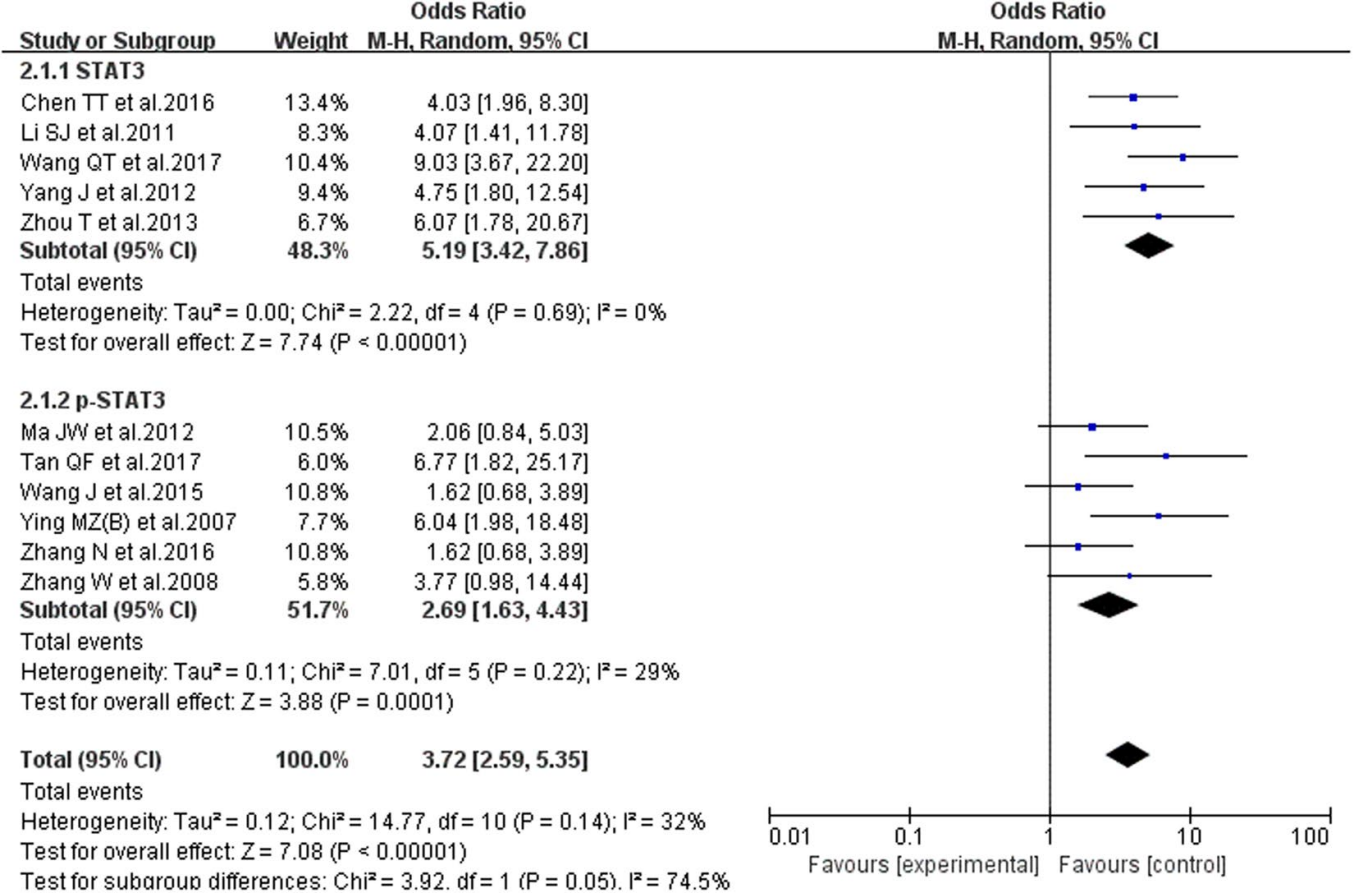
Forest plot of Correlation between STAT3/p-STAT3 expression level and breast cancer lymphatic metastasis. Random-effects OR = 3.72, 95% CI = 2.59–5.35, P = 0.14, I² = 32%.

11 studies reported the correlation of STAT3/p-STAT3 expression level and breast cancer lymphatic metastasis, including 1249 patients of lymphatic metastasis and 350 patients of normal condition. The random-effects model was used and no evident heterogeneity inside (P = 0.14, I^2^ = 32%). The results showed that the STAT3/p-STAT3 expression level of lymphatic metastasis patients is evidently higher than that for other patients (OR = 3.72, 95% CI = 2.59–5.35). In the subgroup analysis, we achieved a consistent result (STAT3: OR = 5.19, 95% CI = 3.42–7.86; p-STAT3: OR = 2.69, 95% CI = 1.63–4.43). The results are shown in Fig. 5.

### Publication bias

**Figure 6:**
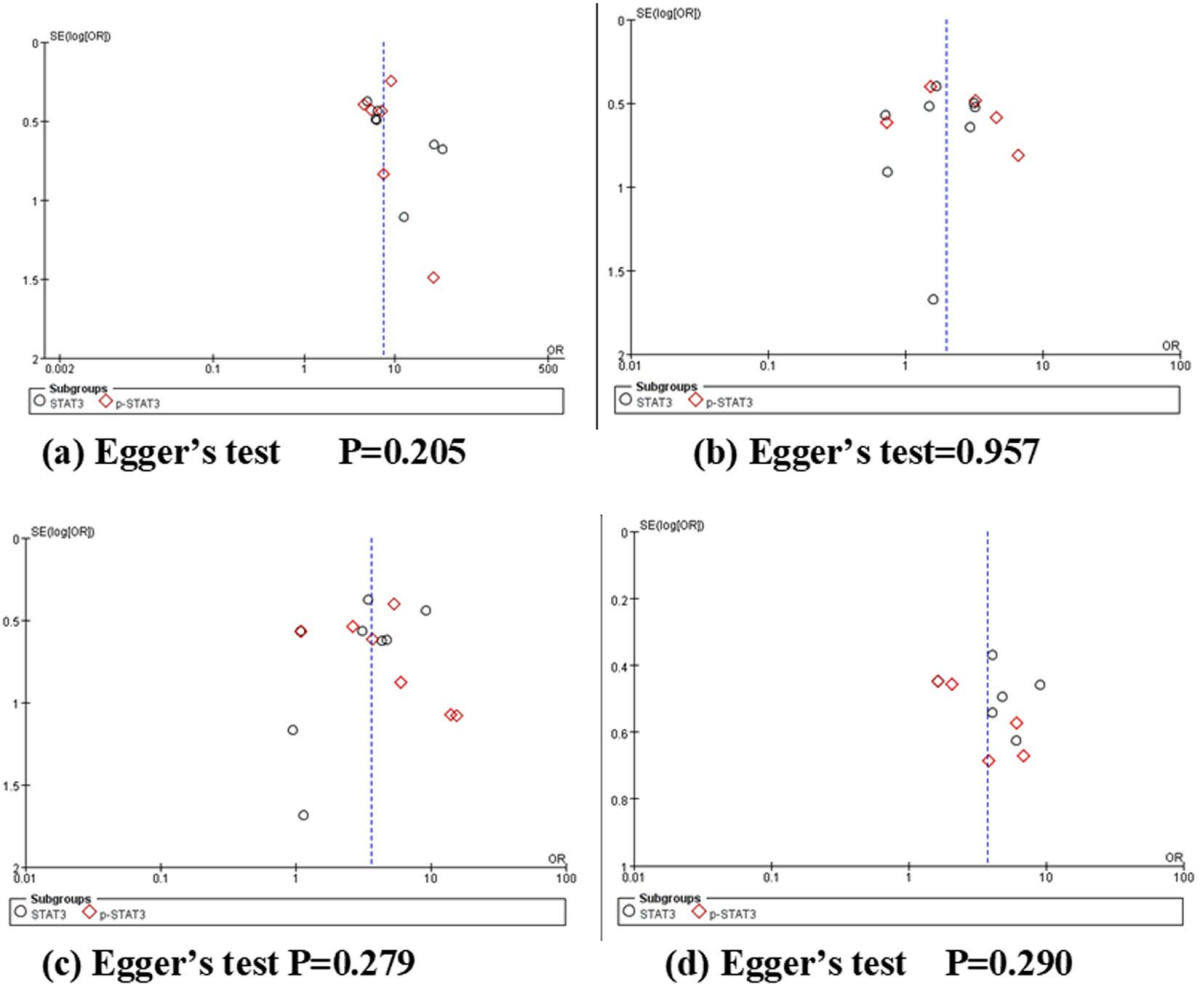
Funnel plots and Egger’s test for publication bias. (**a**) Breast cancer occurrence, (**b**) histological differentiation, (**c**) TNM stages, (**d**) lymphatic metastasis.

Egger’s test performed in STATA 14.0 and funnel plots performed in RevMan 5.3 were used to assess the publication bias of inclusive researches. 18 studies were taken into research, with the funnel plot being shown in Fig. 6, which indicated that there was no obvious publication bias in these studies.

### STAT3/p-STAT3 expression and survival prognosis

To further study the relationship between STAT3 (or p-STAT3) expression and patients’ survival prognosis, five studies were used.

Considering the studies on the relationship between STAT3/p-STAT3 and survival prognosis of breast cancer patients are rare, and the papers did not use a uniformed statistical method, it’s very difficult to generate statistical graphs. The results were shown in the Table 3.

**Table 3:** Studies on the prognostic of STAT3/p-STAT3 in breast cancer.

Reference	Patient numbers	Follow-up(years)	STAT3/p-STAT3	outcome
Li SJ *et al*.^38^	67	8	STAT3	associated with reduced OS
Zhang N *et al*.^41^	91	13	p-STAT3	no significant correlation with OS
Xu S *et al*.^44^	80	5	STAT3	associated with reduced OS
Wang QT *et al*.^50^	130	10	STAT3	associated with reduced OS
Sheen-Chen *et al*.^53^	102	5	STAT3& p-STAT3	associated with reduced OS

p-STAT3: phosphorylated STAT3; OS: overall survival.

## Discussion

JAK-STAT signal pathway is of prime importance for STAT3 phosphorylation^10^. When receptors are stimulated by some special cytokines or growth factors, tyrosine kinase (JAKs) and Src tyrosine kinase coupled with these receptors would phosphorylate STAT3. Moreover, some environmental factors like smoke and UV radiation also phosphorylates STAT3 through tyrosine kinases like Src and ABL independently of receptors^5^.

The gene expression products controlled by STAT3 have multiple functions, like the growth and proliferation of cells, angiogenesis and immunosuppression. P-STAT3 could improve the occurrence of cancers by inducing different kinds of genes controlling cell proliferation to express abnormally. MYC^11^, cyclin D1/D2^12^, BCL-XL^13^, MCL1^14^, surviving^15–17^ and p53^18^ gene expression could improve cell growth and proliferation; VEGF^19,20^, HGF^21^, bFGF^22^, HIF1α^23,24^, MMP2^25^, MMP9^26^, IL-12^27–29^, IFNβ^27,30^, IFNγ^31^, CXCL10^30^, p53^18^ and AKT^23^ gene expression could improve angiogenesis; IL-6^28^, IL-10^32,33^, TGFβ^32,34^, VEGF^19,20^, IFNβ^27,30^, IFNγ^31^, IL-12^27,29^, TNF^27,28^, CXCL10^27^, CCL5^27^, MHC class II^27,31^, CD80^27,31^ and CD86^27,31^ gene expression could induce immunosuppression.

Currently, abundant studies have reported that the STAT3 or p-STAT3 expression has close connection with the occurrence, differentiation, TNM stages and lymphatic metastasis of breast cancer. However, on the one hand, simplex research samples are scarce and have no statistical significance; on the other hand, the results of each research are different. So we performed this meta-analysis to search and screen researches which are satisfactory, and to make our analysis statistically significant.

The breast cancer patients in our research were Chinese. The results showed that STAT3 or p-STAT3 expression in breast cancer tissues was much higher than that in normal ones, indicating a positive correlation between STAT3 or p-STAT3 overexpression and the occurrence of breast cancer. In addition, our research found a higher STAT3 or p-STAT3 expression level in breast cancer cells which kept the characteristics of rapid proliferation, less differentiation and lymphatic metastasis. The STAT3 expression difference has not been found between patients of different ages or tumor sizes. Above all, STAT3/p-STAT3 expression could induce the occurrence of breast cancer; in breast cancer cells, STAT3 or p-STAT3 overexpression could also predict rapid proliferation, the late stage of TNM and the possibility of lymphatic metastasis.

As for survival the relationship between STAT3 (or p-STAT3) expression and patients’ survival prognosis, the outcomes of five studies in Table 3 were not in full accord, but most studies showed the trend that the overexpression of STAT3 (or p-STAT3) was associated with reduced OS, indicating the expression of STAT3/p-STAT3 plays a prognostic role in Chinese breast cancer patients.

There are still many limitations to our analysis. First of all, the inclusive researches are mainly focused on the patients in China, with insufficient persuasion for more massive ethnic groups. Secondly, the difference of inclusive research quality could also affect the reliability of our analysis. Thirdly, the operation methods and evaluation criteria were different in inclusive researches, bringing the potential indeterminacy.

In conclusion, the occurrence of breast cancer has a close correlation with STAT3/p-STAT3 overexpression and phosphorylation. Also, the STAT3/p-STAT3 expression level in tumor tissue could indicate the deteriorating condition, meaning that STAT3/p-STAT3 could be an important target for various cancers. More studies remain to be undertaken for the target STAT3/p-STAT3 protein.
